# Influence of social contact patterns and demographic factors on influenza simulation results

**DOI:** 10.1186/s12879-016-1981-5

**Published:** 2016-11-07

**Authors:** Ruprecht Schmidt-Ott, Markus Schwehm, Martin Eichner

**Affiliations:** 1 GlaxoSmithKline Vaccines, Wavre, Belgium; 2 ExploSYS GmbH, Otto-Hahn-Weg 6, 70771 Leinfelden-Echterdingen, Germany; 3 Department for Clinical Epidemiology and Applied Biometry, University of Tübingen, Silcherstr. 5, 72076 Tübingen, Germany; 4 Epimos GmbH, Uhlandstr. 3, 72144 Dusslingen, Germany

**Keywords:** Influenza, Vaccination, Simulation, Mathematical model, POLYMOD, 4Flu

## Abstract

**Background:**

The demographic composition and the frequency and nature of social contacts may affect the spread of influenza virus in a population, resulting in distinct age-dependent immunity patterns. As demography and social contact rates differ strongly between European countries, this may impact infection incidence and vaccine effectiveness and thus limit the extent to which conclusions derived from observations in one country can be generalized to others. In the current study, we aimed to decipher the impact of social contact patterns and demographic factors on simulation results and, thus, to determine to what extent vaccination results can be generalized.

**Methods:**

We simulated the transmission of four influenza strains (A(H1N1), A(H3N2), B/Victoria, B/Yamagata) in Belgium, Finland, Germany, GB, Italy, Luxembourg, Netherlands and Poland, using the simulation tool 4Flu. Individuals were connected in a dynamically evolving age-dependent contact network based on the POLYMOD study.

**Results:**

When averaged over 20 years, simulation results without vaccination ranged from annually 20,984 (Germany) to 31,322 infections (Italy) per 100,000 individuals. QIV annually prevented 1758 (Poland) to 7720 infections (Germany) per 100,000. Variability of prevented cases remained high when the country-specific vaccination was replaced by unified coverage, but was reduced considerably if the same demography was used for all countries, or even more so when the same contact matrix was used.

**Conclusions:**

Contact matrix and demography strongly influence the age-dependent incidence of influenza and the success of vaccination. Projecting simulation results from one country to another can, therefore, lead to erroneous results.

**Electronic supplementary material:**

The online version of this article (doi:10.1186/s12879-016-1981-5) contains supplementary material, which is available to authorized users.

## Background

Influenza viruses constantly change and vaccines must be reformulated every year. As a consequence, vaccine efficacy from previous years may not be fully applicable for subsequent years. This problem has been recognized and led the European Medicine Agency (EMA) to draft guidelines for influenza vaccines (EMA/CHMP/VWP/457259/2014) in which they request that vaccine effectiveness (VE) for individual influenza vaccines should routinely be investigated. The epidemiology of influenza should be influenced by the demographic composition of the population and by age-dependent immunity patterns which result from prior influenza waves. The frequency and nature of social contacts is also likely to affect the spread of infections in a population. Contact patterns and rates strongly differ among European countries, and this may limit the extent to which modeling results obtained from one country can be generalized. A population-based prospective survey showed that age dependent contact patterns are highly assortative with age [1]. In all countries, the average contact rates (i.e. the number of contacts per individual per day) strongly varied among age groups, usually peaking for juveniles. Furthermore, contact rates averaged over all ages differ substantially between countries (Italians report the highest, Germans the lowest rates). Many European countries are currently experiencing substantial demographic changes (generally, declining birth rates and increasing life expectancy lead to ageing populations), yet demographic projections indicate that these changes occur at very different pace in the European countries [2]. Specifically, as children and juveniles contribute most to the spread of influenza [3], these demographic changes may further limit extrapolations from one country to others.

We examined the influence of contact patterns and demography on the epidemiology of influenza by using the previously published tool 4Flu which simulates the simultaneous and independent transmission of four influenza strains (A(H1N1), A(H3N2), B/Victoria, B/Yamagata) in a population with demographic turnover [4, 5]. Individuals were connected in a dynamically evolving age-dependent contact network based on the contact structures which were determined in the EU POLYMOD study [1].

Mathematical modeling of infectious diseases can help to assess the impact of preventive interventions such as vaccination and increasingly informs public health decisions in this field. Mathematical models influenced the UK’s decision to extend the annual influenza vaccination program to children [6, 7]. They also supported the recommendations to introduce rotavirus vaccination in the childhood vaccination calendar of Germany and human papilloma virus vaccination in Denmark [8, 9]. Mathematical modeling may also help to optimize strategies for influenza vaccine effectiveness assessment. In the current study, we aimed to decipher the impact of social contact patterns and demographic factors on simulation results and, thus, to determine to what extent vaccination results can be generalized. We used a version of 4Flu which has been extended to allow for using demographic data and contact structures of different countries to explore for all eight European countries which participated in the POLYMOD study the influence of contact pattern and demographic changes on the simulation outcomes.

## Methods

**Fig. 1:**
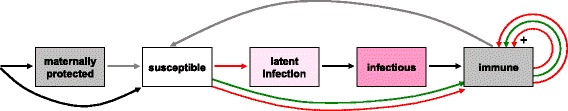
Transmission and immunity dynamics in the simulations: black arrows indicate births and disease progression, red solid arrows indicate infections, green arrows indicate successful vaccinations, and grey arrows show loss of immunity; dotted red arrows indicate cross-immunization against a B lineage caused by an infection or vaccination with the other B lineage; vaccinations and infections can also boost existing immunity (indicated by a “+”); arrows for deaths (which drain each single compartment) were omitted

We used a modified version of the previously published individual-based simulation tool 4Flu [4, 5] which extends the standard susceptible-infected-resistant (SIR) model by including maternal protection, loss of immunity, boosting infections and vaccinations Fig. 1; 4Flu is freely available for download [10]. For a list of modeling parameters, see Additional file 1: Table S1.

### Initialization and evaluation period

Each simulation ran for 40 years: during the first 20 years (starting on July 1st 1994), the age-dependent immunity pattern of the population was initialized by applying trivalent influenza (TIV) vaccinations (using the recorded vaccination coverage and the B lineage which actually was contained in TIV) and allowing for independent transmission of four influenza strains A(H1N1), A(H3N2), B/Victoria and B/Yamagata in populations with demographic turnover. In 2014, a 20 year evaluation period started during which all TIV vaccinations were replaced by vaccination with tetravalent influenza vaccine (QIV) which contained both B lineages. During the evaluation period, the number of cases was reported on a daily basis.

### Demographics

**Fig. 2:**
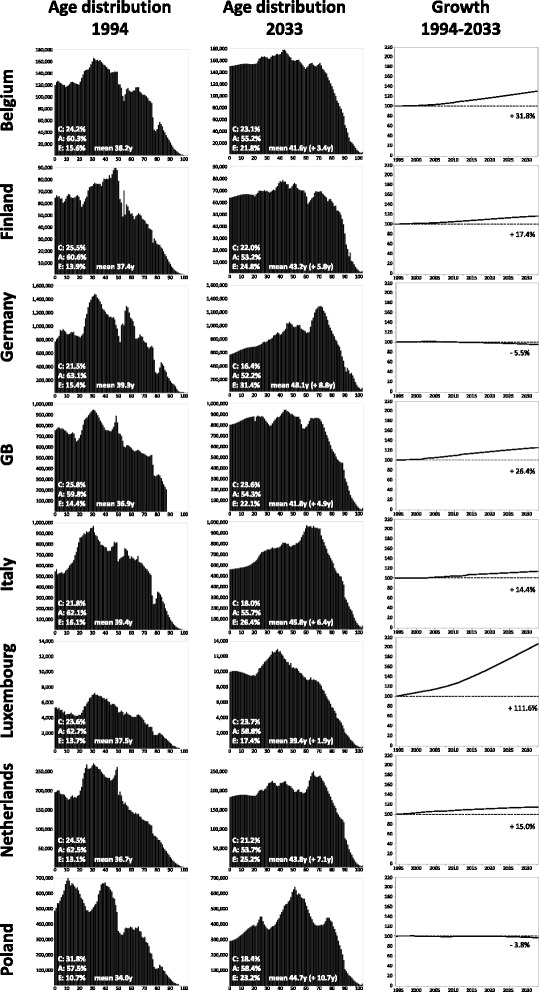
Age distributions of eight European countries. 1^st^ and 2^nd^ column: age distribution of the populations in 1994 and 2033, respectively (the vertical scales of each pair are identical, but they differ between countries). The white inlays give the number of children (C; 0–19 years), young adults (A; 20–64 years) and elderly (E; 65+ years) as well as the mean age in the population and the increase of the mean age from 1994 to 2033. 3^rd^ column: growth of the populations (the size in 1994 is set to 100 %)

We applied this study to each one of the eight countries for which contact structures were determined in the POLYMOD project: Belgium, Finland, Germany, Great Britain, Italy, Luxembourg, the Netherlands, and Poland [1]. For each country, the official demographic age distributions from 1994 to 2033 were used (Fig. 2); data were obtained from Deutsches Statistisches Bundesamt [11] for Germany and from the Eurostat web pages [12] for the other countries. Age-dependent data were complete for the evaluation period (2014–2033), but in some cases, data on very old individuals were missing for various years of the initialization period (1994–2013). Whenever this was the case, the number of individuals in these cohorts was assumed to be zero (i.e. for Italy 1994–2011: 100+; Luxembourg: 1994–2005 and 2007–8: 95+, 2006: 100+; Poland: 1994–2001: 95+, 2002–13: 100+; GB: 1994–2001: 85+; 2002–11: 90+). In the simulations, the initial population size was chosen such that the simulated population reaches exactly 100,000 individuals at the beginning of the evaluation period (on July 1st, 2014). Births, immigrations, deaths and changes of the individuals’ risk status occurred throughout the simulation time, such that the age distribution of the simulated population corresponded exactly to the observed or (for future years) the projected age distribution of the simulated country. Whenever the number of individuals was predicted to increase from 1 year to the next one, new individuals of the appropriate age were created during the simulation (interpreted as immigrants); such “immigrants” were given the infection status and the immunologic status of randomly chosen individuals of approximately the same age [4]. As the vaccination coverage differs for individuals with underlying medical conditions (“at risk” individuals), we had to consider the risk status of individuals. A percentage of newborn individuals was chosen at random to belong to the at risk group. Due to births, ageing and deaths, the age-dependent percentages at risk continuously changed (i.e. they drifted away from the values given in Additional file 1: Table S1). To correct for these deviations, the risk status of randomly chosen individuals were changed throughout the course of the simulations.

### Contact network

Individuals were interconnected in a contact network which changed over time. This network was constructed so that the population statistics of contacts always corresponded to the average number of contacts given by the country’s contact matrix which was derived from the contact matrices reported in the POLYMOD study [1]. The original POLYMOD matrices use 15 categories which usually group individuals in 5-year age classes, and have an asymmetric structure, i.e. they report contacts in an unidirectional way (in an extreme case this structure could imply that an individual of a given age group can contact and infect somebody from another age group, but cannot be infected by the other person himself or herself). We extended the original matrix to a full 101x101 matrix (to explicitly have contact frequencies for any combination of the simulated 101 age cohorts), while keeping the sum of contacts in the 5-year groups identical to the original values. In the simulations, we then applied the extended POLYMOD matrix such that contacts became bidirectional and that the contact distribution evolved as the country’s age distribution changes (see Additional file 1 for details).

### Infection transmission and natural history

Infective individuals passed on the infection to individuals who were in contact with them, but—due to the small basic reproduction number of influenza—usually only a rather small percentage of these contacts were infected. Children have a longer period of infectiousness and can therefore infect a higher percentage of their contact network. The age-dependent average number of contacts between individuals was determined by the country-specific POLYMOD matrix [1] as summarized in Table 1. The infection probability per individual contact per day was calibrated to be 0.03 for the German model by comparing the simulation output with observed data from the 2006/07 German influenza season [4]). In order to be able to compare different countries, this value was used for all countries. In the simulations, it was further multiplied by a seasonal factor which depended on calendar time and which reached a maximum around Christmas [13]. This led to seasonal waves which typically peaked in January or February.

**Table 1:** Summary of demographic and contact features of each country averages over the evaluation period 2014–2033

	Average age [years]	Percentage of population			Contacts per individual per day			
		<20 years	20–64 years	65+ years	<20 years	20–24 years	65+ years	Average
Belgium	41.6	23.0	57.3	19.7	23.2	24.7	13.0	22.0
Finland	43.0	22.1	55.3	22.7	23.6	20.8	8.2	18.5
Germany	46.7	16.9	57.3	25.8	17.1	15.9	9.9	14.5
Great Britain	41.2	23.8	56.6	19.5	26.5	21.8	13.1	21.2
Italy	45.2	18.5	58.2	23.3	50.4	36.2	20.5	35.1
Luxembourg	39.6	23.2	61.3	15.4	38.0	35.8	17.1	33.4
Netherlands	42.8	21.7	57.0	21.3	33.4	27.3	13.0	25.5
Poland	42.7	19.8	60.6	19.6	32.0	33.5	17.7	30.1

### Immunity dynamics

Immunity was lost over time, but could be boosted by later infections. At random years, new drift variants of any one of the four influenza types were introduced which only shared part of their immunologic features with the previously circulating variant which then was replaced by the new one. Thus, the occurrence of drift variants mimicked an additional loss of immunity, leading to an average duration of immunity of 6 years (A(H1N1) and B lineages) or 4.5 years (A(H3N2), respectively, in the absence of boosting events [4]. The two B lineages shared some cross-immunity: infection with one influenza B virus could boost the immunity against the other lineage. Vaccination-derived immunity was lost over time (average duration 1.8 years [4]), but it could be boosted by subsequent infections; vaccinations could also boost infection-derived immunity. The vaccine efficacy depends on the age of the vaccinee [14–16].

### Vaccination

Vaccinations were performed annually in October and November, whereby vaccinees of the previous season were preferentially re-vaccinated in the new season (see Additional file 1: Table S1). The vaccination coverage of all countries, except for the Netherlands, Belgium and Luxembourg, came from Blank et al. [17], using the values for 2007, whereby the values for adults and elderly had to be read from their Fig. 2 using the tool “Data picker” [18]. For the Netherlands, the 2008 vaccination coverage for the total population came from the web pages of the Nationaal Programma Grieppreventie [19]. For Belgium, the 2008 vaccination coverage was calculated from values supplied by the HISIA web pages [20]; to obtain separate values for people with and without risk, we additionally used the percentage of individuals “at risk” from Table 6 of [21]. For Luxemburg, no detailed information of the vaccination coverage could be found. The composition of TIV (which only contains one B lineage) followed the WHO recommendations of past years (1994–2014). Despite containing only one B lineage, TIV vaccination could protect against the missing B lineage or boost pre-existing immunity against the missing B lineage, yet the age-dependent vaccine efficacy of this cross-protection was reduced by 40 % [4]. In some of the years when a new drift variant occurred, the vaccine efficacy was reduced against the new variant (vaccine mismatch).

### Scenario analyses

In a first analysis, each country’s demography was used in combination with its own contact matrix and its own vaccination coverage (Table 2). To obtain a basic set of results, the country-specific vaccination coverage was then replaced by a unified age and risk specific coverage (i.e. by an arbitrarily chosen coverage which was close to the country-specific coverage of most countries): for people without risk status, the vaccination coverage was assumed to be 20 % (0–2 years), 10 % (3–10), 5 % (11–15), 10 % (16–59) and 50 % (60+), respectively; for people with risk status, it was 30 % (0–59) and 70 % (60+), respectively. In two further analyses, we explored how the contact structure and the demography influenced the results by either modifying the contact matrix or the demography while keeping everything else constant.

**Table 2:** Vaccination coverage in the different countries^a^

Country	Group	Vaccination coverage (from age to age) [%]	Ref.
Belgium	not at risk	1.7 (0–17), 6.5 (18–24), 10.1 (25–34), 14.8 (35–44), 18.4 (45–54), 32.5 (55–64), 60.2 (65–74), 72.6 (75+)	[16, 17]
	at risk	5.0 (0–17), 15.3 (18–24), 13.0 (25–34), 15.6 (35–44), 25.2 (45–54), 38.2 (55–64), 60.2 (65–74), 72.6 (75+)	
Finland	not at risk	36.2 (0–2), 6.1 (3–6), 5.1 (7–10), 2.6 (11–14), 6.0 (15–64), 46.5 (65+)	[13]
	at risk	24.0 (0–64), 64.6 (65+)	
Germany	not at risk	19.2 (0–2), 22.4 (3–6), 23.6 (7–10), 11.0 (11–13), 14.4 (14–64), 48.8 (65+)	[13]
	at risk	26.8 (0–64), 76.3 (65+)	
Great Britain	not at risk	13.4 (0–2), 7.1 (3–6), 4.3 (7–10), 3.0 (11–15), 10.6 (16–64), 70.2 (65+)	[13]
	at risk	56.4 (0–64), 91.6 (65+)	
Italy	not at risk	24.5 (0–2), 17.9 (3–6), 14.7 (7–10), 8.3 (11–13), 12.8 (14–64), 56.3 (65+)	[13]
	at risk	42.1 (0–64), 72.7 (65+)	
Netherlands	All	2.0 (0–4), 4.2 (5–9), 4.9 (10–14), 5.0 (15–19), 4.1 (20–24), 4.1 (25–29), 4.7 (30–34), 5.6 (35–39), 8.5 (40–44), 11.2 (45–49), 16.1 (50–54), 30.6 (55–59), 63.9 (60–64), 75.3 (65–69), 81.3 (70–74), 86.1 (75–79), 85.8 (80–84), 85.5 (85–89), 82.7 (90–94), 77.2 (95+)	[15]
Poland	not at risk	10.0 (0–2), 13.0 (3–6), 10.7 (7–10), 5.2 (11–14), 7.5 (15–64), 14.0 (65+)	[13]
	at risk	11.6 (0–64), 17.1 (65+)	
Unified vaccination coverage	not at risk	20.0 (0–2, 10.0 (3–10), 5.0 (11–15), 10.0 (16–59), 50.0 (60+)	
	at risk	30.0 (0–59), 70.0 (60+)	

^a^based on data from 2007 to 2008; sufficient information on Luxembourg was not available

### Reporting

Each simulation was set up to have a population size of 100,000 individuals at the start of the evaluation period. Due to demographic turnover, the simulated number of individuals in earlier years (i.e. in the initialization period) and in later years differed from 100,000. The average annual incidence of influenza infections per 100,000 individuals was calculated by (1) determining the number of infections projected by the model for each evaluation year of each simulation, (2) transforming these numbers into incidence values per 100,000 individuals, (3) averaging the annual incidence values of each simulation over the 20 evaluation years, and (4) by calculating the mean of these values over 1000 simulations. The country-specific number of prevented infections was calculated as the difference between the mean annual incidence values of 1000 simulations without vaccination and with unified vaccination coverage. The country-specific vaccination effect was then given as the percentage of prevented infections.

## Results

### Demographic differences

The age distributions of the eight countries differed widely and they underwent rather extreme changes between the start of the initialization phase (1994) and the end of the evaluation period (2033; Fig. 2), e.g. Poland changed from being the country with by far the highest percentage of children and juveniles (31.8 % below 20 years of age in 1994) to a country which ranked among the lowest (18.4 % in 2033), whereas its percentage of elderly (65+) more than doubled. In each of the eight countries, the population’s mean age increased during the simulation (by at least 1.9 years in Luxembourg up to 10.7 years in Poland). In some countries like Germany, the size of the population declined whereas in others it grew. At least part of this growth must be explained by immigration as is the case for Luxembourg which is predicted to grow by over 100 % until 2033, yet whose age distribution stays nearly constant. The demography of Finland was used in simulations where the same demography was used for every country and where only the contact matrix was varied because its demographic features (percentage of children, average age, annual growth rate) were close to the median of the counties depicted in Fig. 2.

### Differences in contact distributions

**Fig. 3:**
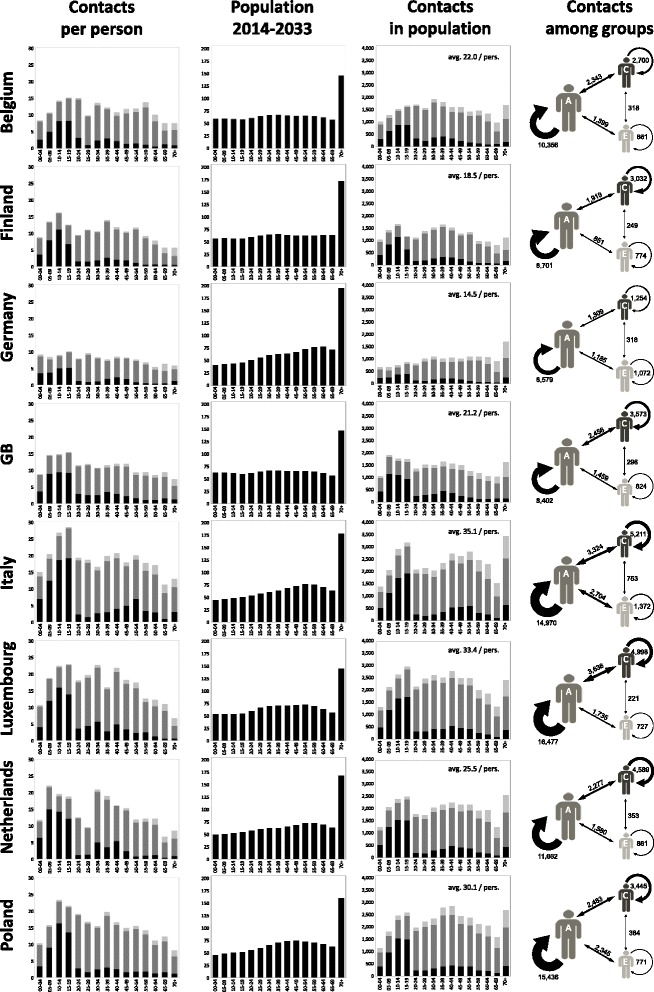
Distribution of contacts for eight countries. 1^st^ column: contacts per individual per day according to the POLYMOD study [5]. Second column: age distributions using the age classes of the POLYMOD study (averages of representative population samples of 1000 individuals per year for the period from 2014 to 2033). 3^rd^ column: total number of contacts per day in a population of 1000 individuals with the age distributions of the 2^nd^ column (combining contacts initiated by the age group and directed to the age group by others; see text for further explanations). Color coding of the bars in the 1^st^ and 3^rd^ column: black = contact with children and juveniles (0–19 years); dark grey: contacts with young adults (20–64 years); light grey: contacts with elderly (65+ years). 4^th^ column: contacts among children (C), young adults (A) and elderly (E); thickness of arrows are proportional to the numbers of contacts (numbers denote daily contacts in a population with a total size of 1000 individuals)

The countries also differed strongly by their numbers of contacts per individual and by the age distribution of these contacts: the 1st column of Fig. 3 shows the results of the POLYMOD study [1]; black sections denote contacts with children and juveniles (0–19 years), dark grey ones contacts with young adults (20–64 years) and light grey ones contacts with elderly (65+ years). The contact matrix of Belgium was used in simulations where the same matrix was used for every country and where only the demography was varied because this contact matrix appeared to lie between the matrices of the matrices of all the counties depicted in Fig. 3. In the simulation tool 4Flu, the contacts depicted in the first column are interpreted as “outgoing” contacts which are initiated by the individuals whose ages are given on the horizontal axis. To obtain the age distribution of all contacts in the population (shown in the 3rd column of Fig. 3), the contacts per individual must be combined with the age distribution of the population (2nd column of Fig. 3; see Additional file 1 and [4] for more explanations). Germany had by far the lowest average number of contacts less than half of the value of Italy, which was the other extreme. The 4th column of Fig. 3 summarizes the contact distribution which prevailed during the evaluation period of the simulations (figure areas are proportional to population size and arrow thickness is proportional to number of contacts): young adults (A) form the largest section of the population; the vast majority of contacts are among young adults, followed by contacts among children (C) and between children and adults. The epidemiological importance of children is further enhanced by the fact that they enter the population susceptible (at least after having passed through a short-term period of material protection) and that they are assumed to have a longer period of infectiousness (Additional file 1: Table S1). Although elderly (E) also form a big segment of the population, contacts among them or between elderly and others are comparatively scarce.

### Results without vaccination

**Fig. 4:**
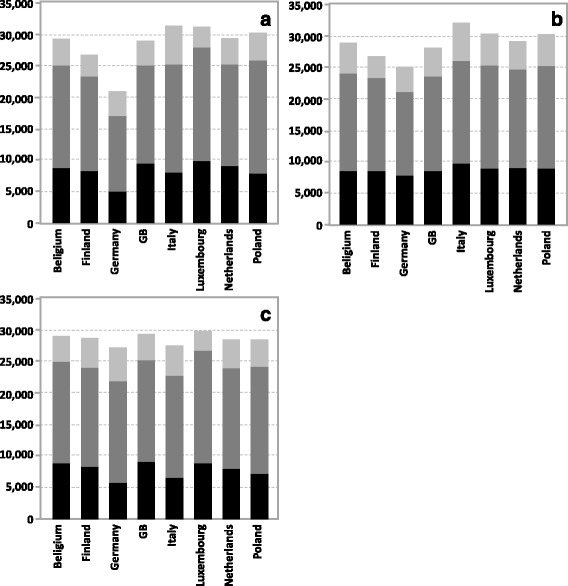
Simulation results for the annual incidence of influenza infections per 100,000 individuals without vaccination in eight countries (black: children (C) 0–17 years, dark grey: adults (A) 18–64 years; light grey: elderly (E) 65+ years). **a** original combination of each country’s demography and contact matrix (coefficient of variation [CV] for C: 17.8 %, A: 12.3 %, E: 20.5 %, all: 11.8 %); **b** combining the Finnish demography with each country’s contact matrix (CV for C: 6.5 %, A: 7.0 %, E: 16.5 %, all: 7.8 %); **c** combining each country’s demography with the Belgian contact matrix (CV for C: 15.3 %, A: 4.1 %, E: 14.6 %, all: 3.2 %). Comparing the variability in the three graphs (either by age group or for the total), using Brown-Forsythe-Test, yielded non-significant results (*p* > 0.05). For each set of simulation parameters, averages of 1000 simulations running for 20 years were calculated

Simulation results without vaccination for the annual influenza infection incidence of the eight countries differed considerably (Fig. 4a), ranging from 20,984 infections per 100,000 per year in Germany to 31,322 infections in Italy. The deviation of the individual countries from their common mean value was quite high (coefficient of variation CV = 11.8 %). Although clearly visible, the differences in inter-country variances were not statistically significant which presumably was due to the small number of countries on which these comparisons were based (Brown-Forsythe test; *p* > 0.05).

### Results of scenario analyses

To examine the influence of the demography on infection incidence, we ran simulations where the countries’ demography was replaced by the Finish demography (Fig. 4b). This had a unifying effect on the results (CV = 7.8 %), much of which was due to infection incidence in children and juveniles 0–19 years of age (whose CV was reduced from 17.8 to 6.5 %) and adults 20–64 years of age (whose CV was reduced from 12.3 to 7.0 %), indicating that these age groups contributed more to the inter-country variability of incidence than the elderly (whose CV only changed from 20.5 to 16.5 %). To also examine the influence of the contact matrix on infection incidence, we ran simulations where each country’s contact matrix was replaced by the Belgian one (Fig. 4c). The unifying effect of replacing the contact matrix was even stronger (CV = 3.2 %) than that of using the same demography. The unifying effect was most pronounced in adults (whose CV is reduced from 12.3 to 4.1 %) and much less in Children (whose CV was reduced from 17.8 to 15.3 %) and elderly (whose CV was reduced from 20.5 to 14.6 %).

### Prevented infections

**Fig. 5:**
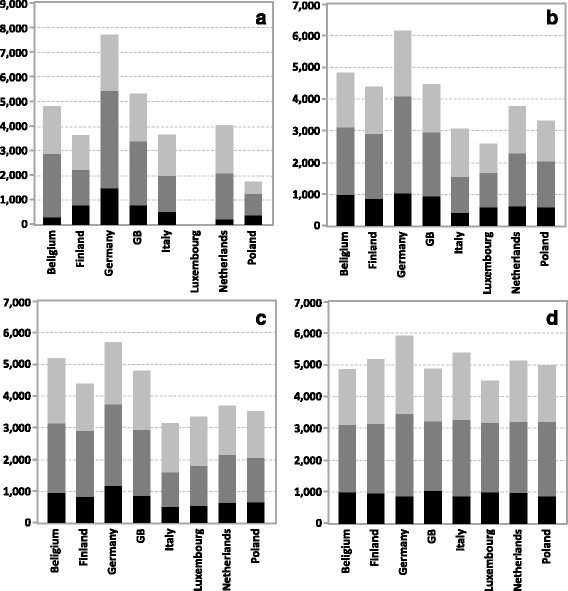
Simulation results for the number of influenza infections which are annually prevented by QIV vaccination in a population of 100,000 individuals (black: children (C) 0–17 years, dark grey: adults (A) 18–64 years; dark grey: elderly (E) 65+ years). **a** combining each country’s specific vaccination coverage with its demography and contact matrix (coefficient of variation [CV] for C: 65.2 %, A: 47.7 %, E: 36.2 %, all: 41.4 %); **b**–**d** using the same unified vaccination coverage for all countries: **b** combining each country’s demography with its contact matrix (CV for C: 31.1 %, A: 34.9 %, E: 22.1 %, all: 27.9 %); **c** combining the Finnish demography with each country’s contact matrix (CV for C: 30.1 %, A: 30.0 %, E: 14.2 %, all: 22.6 %); **d** combining each country’s demography with the Belgian contact matrix (CV for C: 8.6 %, A: 7.2 %, E: 17.9 %, all: 8.0 %). Comparing the variability in the four graphs (either by age group or for the total), using Brown-Forsythe-Test, yielded non-significant results (*p* > 0.05). For each set of simulation parameters, differences are based on 1000 simulations with vaccination and 1000 simulations without vaccination whereby each simulation ran for 20 years

If each country’s own vaccination coverage was used in the simulations, QIV vaccination prevented from 1758 infections (in Poland) to 7720 infections (in Germany; Fig. 5a; Table 3). The CV of the inter-country variability was 41.4 %. Replacing the country-specific vaccination coverage by a unified vaccination coverage (Fig. 5b) reduced the variability to CV = 27.9 %. To allow for a better comparison of countries and to also include Luxembourg (for which the original vaccination coverage was missing), we used the results with unified vaccination coverage in the following analyses. Replacing each country’s contact matrix by the Belgian one (Fig. 5d) again had a large unifying effect on the absolute number of vaccine prevented infections (CV reduced to 8.0 %). The additional unifying effect was most pronounced in children and juveniles (whose CV was reduced from 31.1 to 8.6 %) and younger adults (whose CV was reduced from 34.9 to 7.2 %), but much less in elderly (whose CV was reduced from 22.1 to 17.9 %). Applying the Finish demography for all countries hardly led to any reduction in overall variability of vaccine effects (Fig. 5c; CV = 22.6 %).

**Table 3:** Mean number of infections per year per 100,000 inhabitants

Country	Original demography and contact matrix			Demography of Finland		Contact matrix of Belgium	
	No vacc.	Original vacc.	Unified vacc.	No vacc.	Unified vacc.	No vacc.	Unified vacc.
Belgium	29,194	24,391 (−16.5 %)	24,301 (−16.8 %)	28,799	23,566 (−18.2 %)	29,194	24,301 (−16.8 %)
Finland	26,675	23,002 (−13.8 %)	22,270 (−16.5 %)	26,675	22,270 (−16.5 %)	28,799	23,566 (−18.2 %)
Germany	20,984	13,263 (−36.8 %)	14,795 (−29.5 %)	24,893	19,174 (−23.0 %)	27,249	21,327 (−21.7 %)
Great Britain	28,952	23,617 (−18.4 %)	24,480 (−15.4 %)	27,975	23,121 (−17.4 %)	29,474	24,552 (−16.7 %)
Italy	31,322	27,643 (−11.7 %)	28,238 (−9.8 %)	31,997	28,880 (−9.7 %)	27,584	22,135 (−19.8 %)
Luxembourg	31,216	n.a.	28,588 (−8.4 %)	30,256	26,913 (−11.0 %)	29,970	25,409 (−15.2 %)
Netherlands	29,351	25,319 (−13.7 %)	25,562 (−12.9 %)	29,135	25,437 (−12.7 %)	28,507	23,339 (−18.1 %)
Poland	30,056	28,297 (−5.9 %)	26,750 (−11.0 %)	30,167	26,641 (−11.7 %)	28,498	23,491 (−17.6 %)
CV	11.8 %	21.1 %	18.1 %	7.8 %	12.6 %	3.2 %	5.6 %

Mean number of infections per year per 100,000 inhabitants without or with vaccination, using either the country’s own combination of contact matrix and demography, or replacing the country’s contact matrix by the Belgian one, or replacing the country’s demography by the Finish one; “Original vacc.” denotes the vaccination coverage which actually is used in the different countries (unknown for Luxembourg); “Unified vacc.” uses the same vaccination strategy in every country (see text for details); trivalent influenza vaccine (TIV) is used before 2014; tetravalent influenza vaccine (QIV) is used from 2014 to 2033. Each cell gives the average result of 1000 simulations running from 2014 to 2033

## Discussion

Measuring epidemiologic variability among countries allows investigation of the influence of the contact matrix and demography on infection incidence and vaccine prevented infections. When using the same contact matrix for each country, overall variability of infection incidence was greatly reduced. In contrast, if the same age-distribution was used for each country, this had only a minor harmonizing effect. Thus, the contact matrix was the most important factor which determined differences in country-specific infection incidence. The impact of the contact matrix on vaccine prevented infections was particular striking in children, juveniles and young adults which may be due to the high inter-country differences in the number of contacts of young people (Fig. 3). Thus, when applying the same vaccination strategy in countries with different contact matrices, the number of vaccine prevented infections may differ greatly—particularly for the younger age groups—even if exactly the same age and risk group specific vaccination coverage is used.

As is the case with every modeling study, we had to make some simplifying assumptions. We used the same transmission probability irrespective of the country. Although the same contact questionnaire was used in all POLYMOD countries, contacts among people or reporting could be different between countries. For the sake of examining the influence of contact matrix and age-distribution on the effects of influenza vaccination, it seems justified to keep as many parameters constant as possible, but for a more in-depth evaluation of the benefits of influenza vaccination, it may be warranted to separately calibrate the transmission probability and to use as many country-specific parameter values as possible. We used the same age-dependent percentage of individuals at risk for all countries, whereas the definition of who is regarded to be “at risk” may differ between countries. This should not have a large impact on the results, as the values for Belgium and for the Netherlands tend to be rather similar to the German ones [21–23]. The results for Germany reported in this paper slightly differ from the previously reported ones [4]. Reasons for this are (a) the initialization period and the evaluation period start 1 year later than in the previous paper, (b) the composition of TIV could now be fixed until 2014, and (c) we have extended the number of contact matrix age groups (but this had negligible impact on the results; see Additional file 1 for details). Furthermore, the results in the published paper were given for 0.1 % of the German population whereas here we report results per 100,000 individuals to facilitate comparisons between countries.

## Conclusions

Taken together, the country’s contact matrix and to a much lesser degree the demography influence infection incidence and vaccine effects. The effects vary by age group; for vaccine effects, contact matrices affect more the younger and demography more the older age groups. Projecting simulation results from one country to another can, therefore, lead to erroneous results.

## Additional file

Additional file 1: The online available file “Supporting_Material_4Flu.pdf” explains (1) the extension and smoothing of the original POLYMOD matrix (2) the translation of the resulting matrix into a contact network (3) the occurrence of super-spreaders in the simulation tool 4Flu (PDF 930 kb)

## Abbreviations

EMA: European Medicine Agency
QIV: Quadrivalent influenza vaccine
TIV: Trivalent influenza vaccine
VE: Vaccine effectiveness
