# Supplementary material for: Influence of social contact patterns and demographic factors on influenza simulation results
Source: BMC Infect Dis. 2016 Nov 7;16:646. doi: 10.1186/s12879-016-1981-5 (PMC5100331; doi:10.1186/s12879-016-1981-5)
Supplement: Additional file 1: — The online available file “Supporting_Material_4Flu.pdf” explains (1) the extension and smoothing of the original POLYMOD matrix (2) the translation of the resulting matrix into a contact network (3) the occurrence of super-spreaders in the simulation tool 4Flu (PDF 930 kb) [file 12879_2016_1981_MOESM1_ESM.pdf]

Influence of social contact patterns and demographic factors on influenza simulation results (Supporting material)

Ruprecht Schmidt-Ott[1]
Email: ruprecht.r.schmidt-ott@gsk.com
Markus Schwehm[2]
Email: markus.schwehm@explosys.de
Martin Eichner[3,4]*
Email: martin.eichner@uni-tuebingen.de

[1] GlaxoSmithKline Vaccines, Wavre, Belgium
[2] ExploSYS GmbH, Otto-Hahn-Weg 6, 70771 Leinfelden-Echterdingen, Germany
[3] Department for Clinical Epidemiology and Applied Biometry, University of Tübingen, Silcherstr. 5, 72076 Tübingen, Germany
[4] Epimos GmbH, Uhlandstr. 3, 72144 Dusslingen, Germany

* Corresponding author. Department for Clinical Epidemiology and Applied Biometry, University of Tübingen, Silcherstr. 5, 72076 Tübingen, Germany

Previous work

This paper extends a previous manuscript which examined the impact of tetravalent influenza vaccination (QIV) in Germany [1]. The parameter values which are used in the current paper (which are mostly identical to those in the first paper) are given in Table S1.

Extension of the POLYMOD matrix

The original POLYMOD matrices reported the daily numbers of contacts between individuals of different age groups (0-4, 5-9, 10-14, …, 65-69, 70+ years) [2]. A description of the construction of a contact network based on these matrices was published by Eichner *et al.* [1]. Detailed examinations showed that the five-year age groups of the POLYMOD matrix on which the contact structure was based, led to artificial patterns in the detailed age-distribution of cases (results not shown). Although these artificial structures are averaged out if the results are aggregated in large age groups (as is the case in this paper and as was the case in the original publication of Eichner *et al.* 2014), it was decided to extend the contact matrices from their original 15x15 age groups to the 101x101 age cohorts in order to obtain realistic results in our subsequent economic analyses [3] which strongly depended on age-dependent outcomes. This extension was done such that all the information contained in the original POLYMOD matrices is retained, as will be shown below. We started with a POLYMOD matrix P_{c_1,c_2} which gave the daily number of contacts of individuals of age group c_1 with individuals of age group c_2 (c_1 and c_2 typically were five-year age classes; cf.). If we simply extended this to a matrix $M_{i,j}$ with 101x101 entries by appropriately copying the existing 15x15 values of the POLYMOD matrix into a 101x101 matrix (cf. Figure S1) we would create too many contacts (e.g. a ten year old child would now have the same number of contacts with 20 year olds, 21 year olds, …, 24 year olds as the child should have with all individuals together in the age group 20-24). To correct for this, we distributed the number of contacts according to the number of individuals in the target age cohorts. This was done by the multiplication $M_{i,j} \cdot N_j / N_{c(j)}$ whereby N_j was the number of individuals who are j years old, and $N_{c(j)}$ was the number of all individuals in the POLYMOD age group to which cohort j belonged (for this, we used the demographic data of the year 2006 in which the POLYMOD data were collected). The matrix $E_{i,j}$ which was the result of this multiplication had exactly the same number of contacts as the POLYMOD matrix when re-aggregating the age-groups (i.e.

it appropriately extended the original POLYMOD matrix), but it still showed the same abrupt changes every five years as the original matrix. This problem can, for example, be illustrated by looking at the contacts of an individual of age i with other individuals of age $i+1$: using the simple extension of the POLYMOD matrix as described above, these contacts were controlled by the first sub-diagonal of the extended contact matrix which passed through a block of the diagonal the original POLYMOD matrix for four years and then through a block of the sub-diagonal of the original POLYMOD matrix for one year. Since the diagonal elements in the POLYMOD matrix have higher values than the elements on the sub-diagonal, the probability of contacts between two individuals periodically had to change from a high value (four years) to a low value (one year). The contact network of the simulator, therefore, had to periodically establish and remove contacts due to the artificial 5 years granularity of the POLYMOD matrix while individuals were ageing. Similar arguments hold if the individuals' ages are further apart. To remove this artificial change of contact probability every time a pair of individuals crosses the 5x5 cohort boundaries, the extended contact matrix needed to be smoothed in particular in the direction every pair of individuals moves by aging. In order to get rid of this feature and of the associated artifacts, we smoothed the extended contact matrix by imposing a curvature measure. A variety of functions to compute the curvature of the matrix has been considered: from computing the curvature in the direction of the main diagonal to computing the curvature equally in all directions (horizontal, vertical and both diagonals). The curvature function chose here emphasizes the curvature in the direction of the main diagonal (produced by ageing), by multiplying with a factor of two. The curvature term for the opposite diagonal was deliberately omitted, because this term would also smooth and, thereby, diminish the "rift" along the main diagonal, representing contacts between individuals of the same age. Such a rift has been observed in other contact studies addressing children at school, where it was observed that these contacts were highly assortative by age [4]. In technical terms, we assessed the smoothness of the surface of the matrix $E_{i,j}$ at the location i,j by calculating the curvature measure

$$\begin{aligned} D_{i,j} = & \left(\left(E_{i-1,j} - E_{i,j}\right) - \left(E_{i,j} - E_{i+1,j}\right)\right)^2 \\ + & \left(\left(E_{i,j-1} - E_{i,j}\right) - \left(E_{i,j} - E_{i,j+1}\right)\right)^2 \\ + & 2\left(\left(E_{i-1,j-1} - E_{i,j}\right) - \left(E_{i,j} - E_{i+1,j+1}\right)\right)^2. \end{aligned}$$

This calculation took into account the increase or decrease in values on three axes: horizontally (1st term), vertically (2nd term) and diagonally (3rd term), using six of the eight neighboring fields. As it was important to have a very smooth curve along the diagonal axis which corresponds to ageing (while individuals age from i-1 to i to i+1 years, their contacts age from j-1 to j to j+1 years) the curvature on this diagonal axis was penalized by using the factor 2, whereas the curvature on the opposite diagonal axis was ignored; for combinations of i and j at the edge of the matrix, some relations to neighbor cohorts cannot be evaluated and their terms were set to 0. If the number of contacts increases or decreases in exactly the same way before and after the position i,j on any one of the 3 axes, the value of $D_{i,j}$ becomes zero and the surface of the matrix is locally perfectly smooth. This was the case in the center of the 5x5 POLYMOD age groups, but certainly not at their edges. In the next steps, we smoothed the matrix $E_{i,j}$ in a way which did not change the total numbers of contacts within and between the (original) five-year age groups. Figure S1 shows the relation of the extended matrix $E_{i,j}$ and the original POLYMOD matrix P_{c_1,c_2}. Any exchange of contacts between fields which belong to the same combination of POLYMOD age groups (typically these are 5x5 squares in Figure S1) leads to a matrix which still is equally compatible with the POLYMOD matrix as the trivially extended matrix $E_{i,j}$. This property was used to smooth the surface of the extended matrix: first we randomly picked a combination (i_1,j_1) of cohorts, e.g. (5,31) shown as a black square in Figure S1. Then, we determined to which POLYMOD age groups

this combination belonged; in our example, it belonged to (5-9,30-34). Next, we picked another random combination of cohorts (i_2,j_2) which now had to come from the same combination of POLYMOD age groups as the first one. This could for example be (8,33), shown as a grey square in Figure S1. Now, we transferred as many contacts from (5,31) to (8,33) (or vice versa) as were needed to locally minimize the smoothness of the surface, i.e. which minimize $D_{i_1,j_1} + D_{i_2,j_2}$. This random sampling and local smoothing was performed for 100,000,000 times before the final smooth matrix $E'_{i,j}$ was obtained which then contained the number of contacts of an individual of cohort *i* with people of cohort *j*.

From contact matrix to contact network

Using a contact matrix to simulate the transmission of an infectious disease may seem straight-forward, but at a closer look, it can create serious interpretation problems. Figure S2 visualizes these problems for an artificial situation where individuals (depicted as circles) belong to two different age groups. Individuals of age group 1 (black circles) claim to have two contacts with individuals of age group 2 (grey circles), whereas those claim to have only one contact with the former. For diseases like influenza, we have to assume bidirectional contacts (meaning: if individual A has a contact with B, B also has a contact with A), yet satisfying the claims of the first age group may easily create a conflict with the claims of the second one as is shown in Figure S2a. Only if the ratio of the two population sizes exactly matches the ratio of their contacts, can both claims be satisfied simultaneously (Figure S2b). This problem was also inherent in the original and in the extended POLYMOD matrices: using for example the original POLYMOD matrix for Belgium, individuals in the age group “20-24” claim to have 1.77 contacts per day with the “25-29” group, whereas those claim to have only 0.91 contacts per day with the former group [2], implying that one group should be twice the size of the other which was in conflict with the demographic data. Even if a contact matrix would be constructed such that it satisfied all these conditions for any given combination of age groups, this would only work until the demographic structure of the population changed. This problem is not restricted to individual-based simulations, but it is equally present in transmission models based on differential equations.

In the simulation tool 4Flu, a straight-forward way of creating bidirectional contact network which is aligned to a given unidirectional contact matrix was achieved by distinguishing between “outgoing” contacts and “incoming” contacts: on average, each individual in the population initiates the number of “outgoing” contacts to individuals of the different age groups as is stated by the POLYMOD matrix, whereas all these “outgoing” contacts are recorded as “incoming” contacts for the individuals who receive these contacts [1]. Figure S2c shows the result of this procedure applied to the example “population” where the members of the first age group claim to have two contacts with the second age group whose members only claim to have one contact with the others. Although the contacts are internally labeled as “outgoing” and “incoming”, both are regarded as bidirectional contacts when it comes to transmitting the infection; the distinction only serves to create a population average of the contact matrix. When simulated individuals aged and when other demographic changes took place, random contacts were removed or added to keep the population statistics of “outgoing” contacts synchronized with the values in the extended POLYMOD matrix at any time. In the absence of conflicts between contact matrix and demographic distribution, the described procedure doubles the total number of contacts in the population, but this is not problematic, because infected individuals only infect a small percentage of their contacts, depending on the immunity of the contacts and on the probability of transmitting the infection to a susceptible contact. Calibrating the simulation model for Germany to obtain a median infection incidence of 10.6% in young adults in the 2006/07 season [5], a transmission probability of 3% per day per contact was obtained. This value was used for all simulations.

Super-spreaders in the 4Flu network

Whereas a random uniformly mixing network would yield a normal distribution of the degree of individual connections which would be centered around the average number of contacts, the degree distribution of the 4Flu network is moderately skewed. This means that a few individuals have much more contacts than others, outweighed by a larger number of individuals with below average contacts, i.e. the 4Flu contact network contains so-called super-spreaders of varying degree. Digging deeper into the network structure reveals that most of these super-spreaders in 4Flu are aged between 5 and 20 years. The degree of these super-spreaders shows that they are only “moderate” super-spreaders, but interestingly, these moderate super-spreaders tend to be interconnected, i.e. the 4Flu network contains clusters of moderate super-spreaders. As an interpretation, we could associate these clusters with school children, yet no assumptions about schools were incorporated in the model, but the network structure was solely derives from the original POLYMOD matrix and the above explained smoothing procedure.

Figures

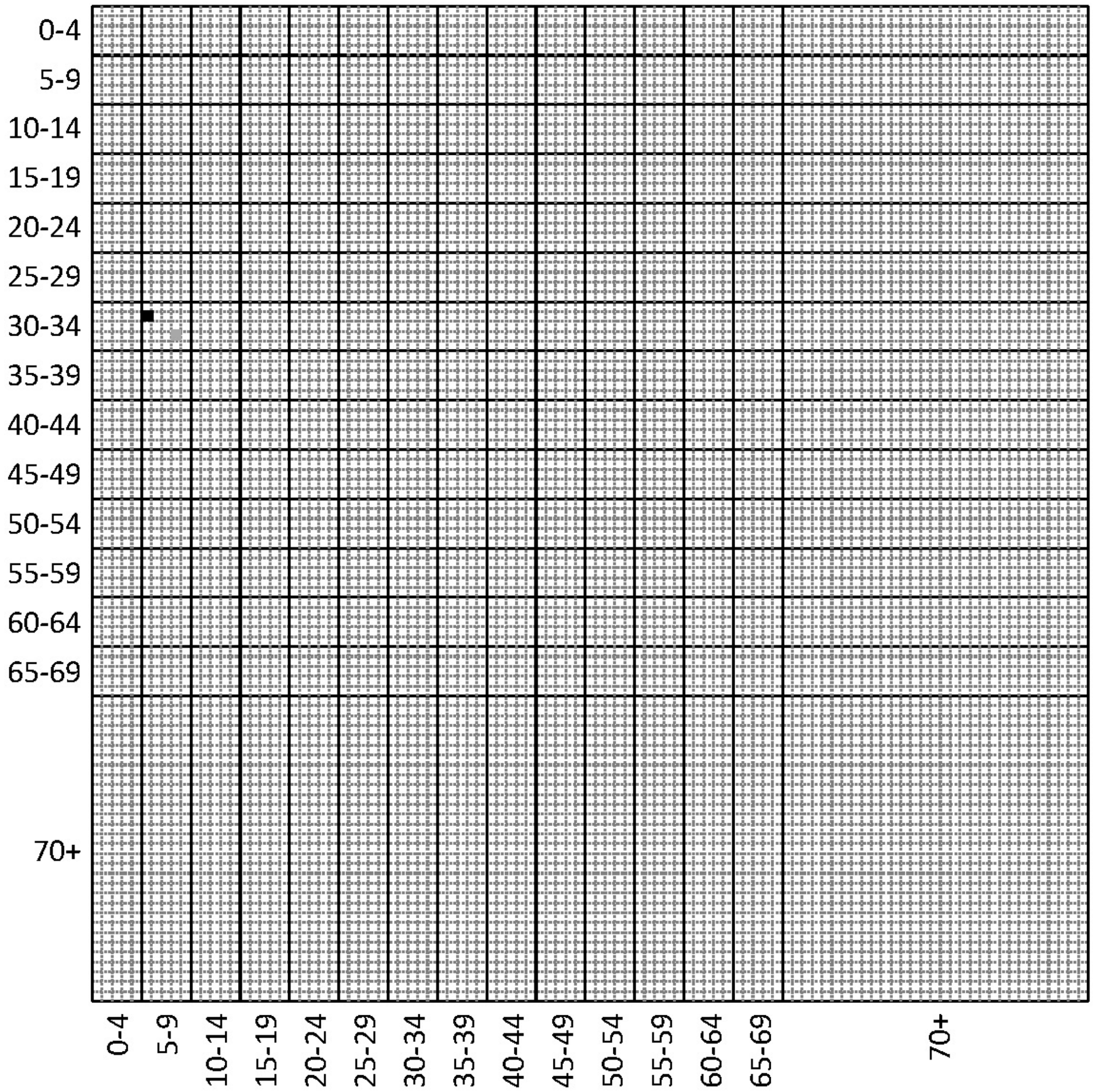

Figure S1 Visualization of the extension of the POLYMOD matrix into a smoothed 101x101 matrix. The POLYMOD matrix typically gives the number of contacts in 5-year age groups (delimited by full lines); e.g. Belgian children of the age group 5-9 on average have 1.29 contacts per day with adults of age group 30-34; retaining the total number of contacts, these contacts are split into 1-year cohorts such that e.g. the number of contacts between 5 year old children and 31 year old adults is given. The two contact rates which are shown as black and grey squares are used in the text to illustrate how the surface of the new contact matrix is smoothed, using randomly picked values.

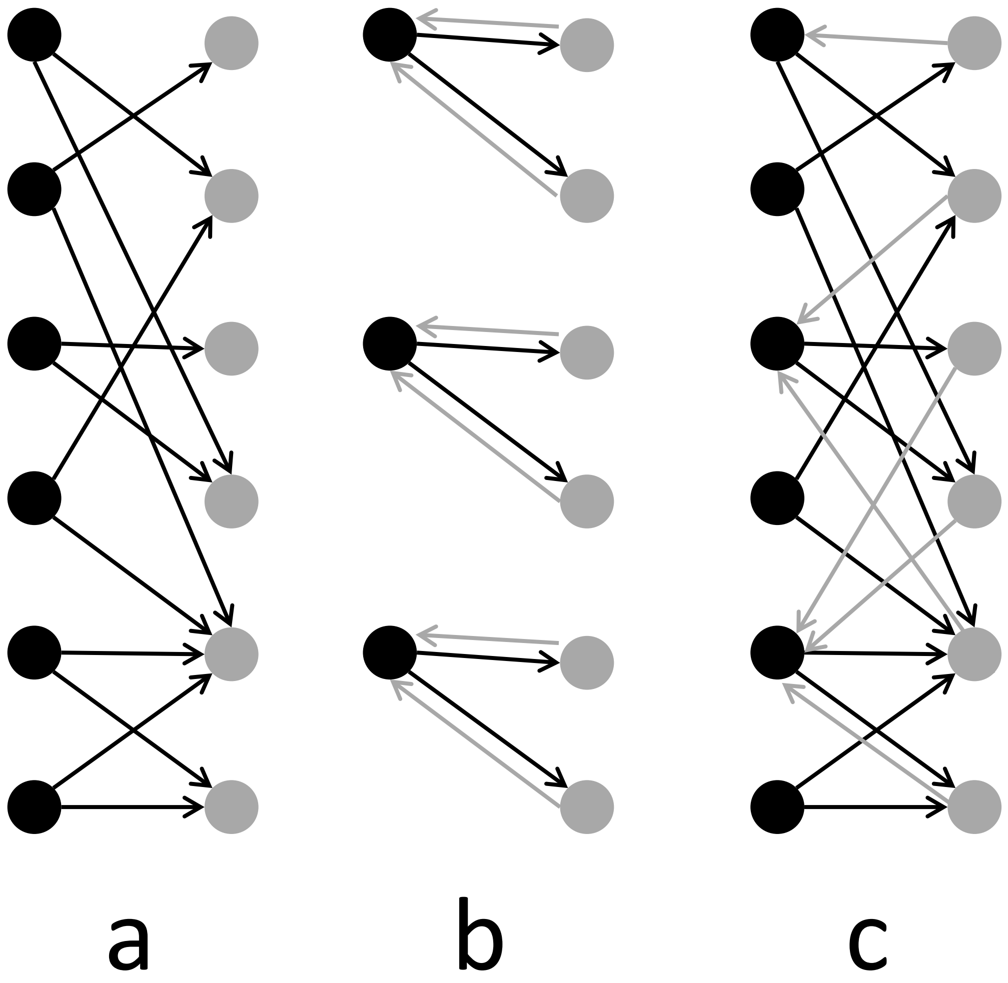

Figure S2 Visualization of the problem with bidirectional (see text of the Online Supporting Material for details). Black and grey circles denote individuals of two different age groups; arrows denote contacts. (a) Unidirectional contacts, (b and c) bidirectional contacts in a population.

Table S1 List of parameters and baseline values.

Parameter	Value	Reference
Infections per day per contact	0.03	calculated from [5]
Maximum seasonal transmission factor	1.43	[6]
Day of maximum seasonal transmission	Dec. 21st	[6]
Duration of the latent period	2 days	[7]
External infection probability	0.0003/year	assumed
Duration of the infectious period<br>- children (age 0–17 years)<br>- adults (age 18 years and above)	 4 days<br>2 days	 [7]
Duration of maternal protection	2 - 4 months	[8, 9]
Immunity loss rate after infection	1 / (9.13 years)	calculated from [6], see [1]
Average circulation time per drift variant<br>- A(H1N1), B/Vic, B/Yam<br>- A(H3N2)	 7.0 years<br>3.5 years	 calculated from [10, 11],<br>see [1]
Revaccination preference factor	2	assumed
Probability of mismatched vaccine design when a new drift variant occurs	40%	calculated from [10-12], see [1]
Vaccine efficacy (well-matched vaccine)<br>- 0–1 year of age<br>- 2–5 years of age<br>- 6–15 years of age<br>- 16–64 years of age<br>- 65 years of age or older	 45%<br>39%<br>69%<br>63%<br>58%	 [13]<br>[13]<br>[13]<br>[14]<br>[15]
Cross protection after infection<br>- percentage of individuals who are immunized against a B lineage when they are infected (or boosted) with the other B lineage (lineage cross protection)<br>- percentage of individuals who were immune against the previous drift variant, who are still protected against the new one (drift cross protection)	 60%<br><br><br><br>60%	 calculated from [16], see [1]
Cross protection after vaccination<br>- vaccine efficacy multiplication factor for B lineage not contained in TIV (lineage cross protection)<br>- vaccine efficacy multiplication factor for vaccinations with drift mismatch (drift cross protection)	 0.6<br><br>0.6	 calculated from [16], see [1]
Immunity loss rate after vaccination	1/(1.8 years)	calculated from [17], see [1]
Percentage of the population with elevated risk<br>- newborn individuals<br>- age 0 to 15 years<br>- age 16 to 59 years<br>- age 60 years and above	 3.0%<br>6.0%<br>14.2%<br>47.1%	 [18]

References

1. Eichner, M., et al., *4Flu - an individual based simulation tool to study the effects of quadrivalent vaccination on seasonal influenza in Germany.* BMC Infect Dis, 2014. **14**: p. 365.
2. Mossong, J., et al., *Social contacts and mixing patterns relevant to the spread of infectious diseases.* PLoS Med, 2008. **5**(3): p. e74.
3. Dolk, C., et al., *Cost-utility of quadrivalent versus trivalent influenza vaccine in Germany, using an individual-based dynamic transmission model.* in press.
4. Conlan, A.J., et al., *Measuring social networks in British primary schools through scientific engagement.* Proc Biol Sci, 2011. **278**(1711): p. 1467-75.
5. Williams, C.J., et al., *Seasonal influenza risk in hospital healthcare workers is more strongly associated with household than occupational exposures: results from a prospective cohort study in Berlin, Germany, 2006/07.* BMC Infect Dis, 2010. **10**: p. 8.
6. Vynnycky, E., et al., *Estimating the impact of childhood influenza vaccination programmes in England and Wales.* Vaccine, 2008. **26**(41): p. 5321-30.
7. Haas, W., ed. *Influenza: Prävention, Diagnostik, Therapie und öffentliche Gesundheit*. 2009, Elsevier GmbH, Munich, Germany.
8. Zaman, K., et al., *Effectiveness of maternal influenza immunization in mothers and infants.* N Engl J Med, 2008. **359**(15): p. 1555-64.
9. Poehling, K.A., et al., *Impact of maternal immunization on influenza hospitalizations in infants.* Am J Obstet Gynecol, 2011. **204**(6 Suppl 1): p. S141-8.
10. Smith, D.J., et al., *Mapping the antigenic and genetic evolution of influenza virus.* Science, 2004. **305**(5682): p. 371-6.
11. Hay, A.J., et al., *The evolution of human influenza viruses.* Philos Trans R Soc Lond B Biol Sci, 2001. **356**(1416): p. 1861-70.
12. Influenza, A.f. *Arbeitsgemeinschaft für Influenza. 2013. Saisonberichte.* 2013; Available from: http://influenza.rki.de/Saisonbericht.aspx.
13. Jefferson, T., et al., *Vaccines for preventing influenza in healthy children.* Cochrane Database Syst Rev, 2012. **8**: p. CD004879.
14. Demicheli, V., et al., *Vaccines for preventing influenza in healthy adults (Review).* Cochrane Database Syst Rev, 2015.
15. Jefferson, T., et al., *Vaccines for preventing influenza in the elderly.* Cochrane Database Syst Rev, 2010(2): p. CD004876.
16. Jefferson, T., et al., *Vaccines for preventing influenza in healthy adults.* Cochrane Database Syst Rev, 2010. **7**: p. CD001269.
17. Ruf, B., et al., *Immunogenicity and antibody persistence of FluarixTM vs. Fluad(R) vs. Inflexal VTM in the elderly.*, in *Poster presented during the First International Conference on Influenza Vaccines for the World*. 2004: Lisbon, Portugal.
18. Institute, R.K. *Robert Koch Institute. 2007. Influenzapandemieplan.* 2007; Available from: http://www.rki.de/DE/Content/InfAZ/I/Influenza/Influenzapandemieplan.html.
